# A MEMS-Based Multi-Parameter Integrated Chip and Its Portable System for Water Quality Detection

**DOI:** 10.3390/mi11010063

**Published:** 2020-01-05

**Authors:** Ziyue Wu, Jiaqi Wang, Chao Bian, Jianhua Tong, Shanhong Xia

**Affiliations:** 1 State Key Laboratory of Transducer Technology, Aerospace Information Research Institute, Chinese Academy of Sciences, Beijing 100000, China; wzy41351107@163.com (Z.W.);; 2 University of Chinese Academy of Sciences, Beijing 100000, China

**Keywords:** multi-parameter, portable system, water quality detection

## Abstract

As an important means to protect water resources, water quality detection is of great social and economic significance. Water quality detection sensors processed by micro-electro-mechanical system (MEMS) technology have the advantages of low-cost, small size, and high sensitivity. In this paper, a multi-parameter water quality detection integrated sensor chip is further studied, and a portable detection system using this chip is developed. Temperature, pH, oxidation-reduction potential (ORP), conductivity and concentration of copper ions (Cu^2+^) are selected as typical water quality parameters. Experiments of sensor calibrations using this portable detection system were performed in standard solutions. The sensor has a sensitivity of −57.34 mV/pH in pH detection and 5.95 Ω/°C in temperature response. ORP is directly detected by Pt microelectrode on the chip and the relative error is less than 3%. The electrode constant of the sensor is 1.416 cm^−1^ and the linearity is 0.9995 in conductivity detection. With the gold nanoparticles deposited on the electrode, the detection peak of Cu^2+^ appears at 280 mV and the sensor shows good linearity to the concentration of Cu^2+^ in the range of 0–0.6 mg/L. The detection limit of Cu^2+^ concentration is 2.33 μg/L. Through measurement and calculation, the accuracy of the portable system is within 4%. This portable multi-parameter water quality detection system with the MEMS-based integrated chip shows great potential in the field and fast detection.

## 1. Introduction

The contamination of water has attracted many people’s attention all over the world. Water protection can start from the water quality inspection through the detection of physical and chemical indicators in the water samples. It can obtain the status of water pollution and also provide an important reference for subsequent treatment decisions [1]. Due to the lack of rapid and effective detection and treatment methods, large-scale water pollution incidents are still occurring repeatedly [2,3]. In the past three decades, more and more researches focus on water quality detection and lots of guidelines and quality standards have been developed by specialized regulators and organizations [1,3]. Traditional water quality detection requires taking water samples back to the laboratory, which takes too much time. As computer and communication technologies increase, the development trend of water detection instruments is low-cost, miniaturization, easy operation, high precision and reliability [1,2,3].

A series of parameters can be used to characterize water quality, including pH, turbidity, free chlorine, dissolved oxygen, conductivity, temperature, oxidation-reduction potential (ORP), heavy metal, and so on [1,4]. The detection of a single parameter is not enough to judge water quality [2]. However, it is impractical to monitor all the parameters in water at the current stage because of the complexity of replacing, regulating and maintenance of the sensor probes [1]. Multi-parameter, integrated and simultaneous detections are the foci of current researches. The appearance and development of micro-sensors make the multi-parameter integrated detection become a reality [2,3,4,5,6,7]. The manufacture of these integrated sensor chips adopts advanced and mature processes such as thick-film technology [5], inkjet printing techniques [2,6] and micro-electro-mechanical system (MEMS) technology [4,8]. For remote and portable monitoring of water quality, Internet of Things technology [3], microcomputer technology [9,10] and wireless communication technology like Wi-Fi [3] and Bluetooth [9] are used frequently.

For the water quality, the temperature is an important factor in the determination of many physical and chemical parameters of water quality, and it can directly affect the measurement results of pH, conductivity and other monitoring items [1,2,5]. Apart from glass liquid thermometers and bimetal thermometers, thermistors are also commonly used. Among them, the resistance temperature detector (RTD) processed from platinum (Pt) is often used because of its good stability and accuracy [4]. As a measure of acidity and alkalinity of solutions, pH can reflect whether water quality is polluted by acid or alkaline substances and indicate the degree of pollution, which is very important to human health [11]. For a multi-parameter integrated sensor chip and its portable system, the traditional glass electrode of pH is not a suitable choice. In previous studies, metal oxide electrodes showed good Nernst response, of which RuO_x_ had the characteristics of low contamination, easy preparation and good chemical resistance [12]. As one of the electrical and physical characteristics of water quality, conductivity reflects the level of electrolytes present in the water and acts as an important indicator of the purity of water [11,12].

ORP monitoring first appeared in the study of chlorine oxidation ability. Its value is related to the disinfectant efficiency of water quality [11]. Currently, ORP detection has been recognized by many international and national health standards. As a drinking water standard, ORP value takes into account varieties of chemical elements in the water, which is easy to display the water quality with electronic instruments. According to the Environmental Protection Agency (EPA), the ORP of drinking water should be approaching 250 mV [13]. ORP is an electrochemical parameter and the principle of an ORP electrode is not obscure. The direct way to measure it is to detect its potential against a reference electrode (RE) [11,14].

Heavy metal ions pollution has become a serious environmental problem. In aquatic ecosystems, heavy metals ions change the fine structure of the plant’s locomotor, inhibit their photosynthesis and respiration, alter the composition of nucleic acids, and affect cell volume [15,16,17]. Excessive levels of heavy metals in animals cause inactivation of enzymes and cytotoxicity, which further affect genetic expression, damage nerve tissue and important immune organs [18,19,20]. The traditional methods for heavy metal ions detection include but are not limited to atomic fluorescence spectrometry, inductively coupled plasma, atomic absorption spectrometry, ultraviolet-visible spectrophotometry [21,22], and electrochemical detection. The electrochemical detection is simple in principle and easy to implement just with a triple-electrode system. A triple-electrode system is composed of a working electrode (WE), a counter electrode (CE) and a RE. By using different materials to modify the working electrode, identification of specific ions can be achieved and detection performance can be improved [15]. For instance, the microelectrodes modified by gold nanoparticles (AuNPs) show satisfied analytical performances in the detection of copper, lead and zinc ions [23].

In this paper, a multi-parameter water quality detection integrated chip is further studied. The previous work about the design, fabrication, and testing results of the temperature, pH and conductivity sensors on this integrated chip has been reported [12]. This paper makes some additions. The new research focuses on the development of ORP and copper ions (Cu^2+^) detection on the integrated sensor chip, and its portable detection system of the above five parameters is designed and developed. This multi-parameter integrated chip is fabricated by MEMS techniques and electrochemical modification technology. The Pt film electrode is prepared to detect the potential of ORP. The copper irons detection sensor adopts an interdigital electrodes structure, with an AuNPs film deposited on its surface to improve the detection capability. In our portable detection system, a piece of STM32F407VGT6 chip is used as the main microcontroller unit (MCU). A 0.96-inch organic light-emitting diode (OLED) screen is used to display data. A Bluetooth chip is connected to the serial port in the MCU, which can transmit data to the computer or other mobile devices to display the data and waveforms. This project aims to develop a portable water quality detection system based on the multi-parameter integrated sensor chip, which can be used to detect parameters including temperature, pH, conductivity, ORP and copper ions in water standards [24,25,26]. In the standards, the pH of drinking water should be between 6.5 and 8.5 at normal temperature [24,25]. The conductivity of general natural water is less than 1.5 mS/cm, while water containing inorganic salts can reach 10 mS/cm [26]. The concentration of copper ions in water should be less than 1 mg/L [24,25]. The experiments with the MEMS sensor chip and its portable system proposed in this project show satisfactory results in terms of miniaturization, accuracy, linearity, and sensitivity. The whole system has great potential for portable, rapid and accurate measurements of actual water samples.

## 2. Materials and Methods 

### 2.1. Multi-Parameter Integrated Sensor Chip

**Figure 1:**
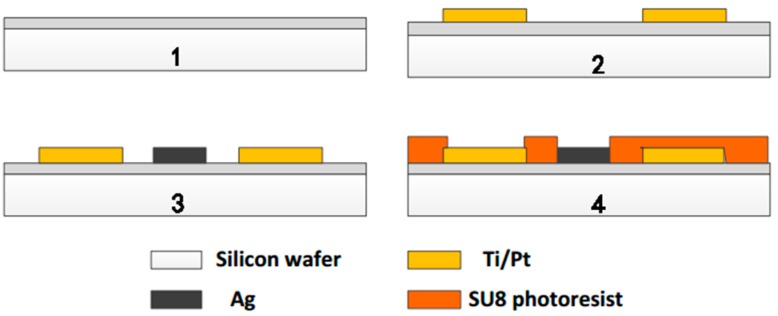
Fabrication process of the integrated microchip.

Considering the good electrical and temperature characteristics, we chose Pt as the substrate metal of the multi-sensor integrated chip [12]. We used 4-inch silicon wafer processing technology and basic MEMS technology to fabricate the structure of each part of the microchip. The simplified fabrication process [12] is shown in Figure 1.

**Figure 2:**
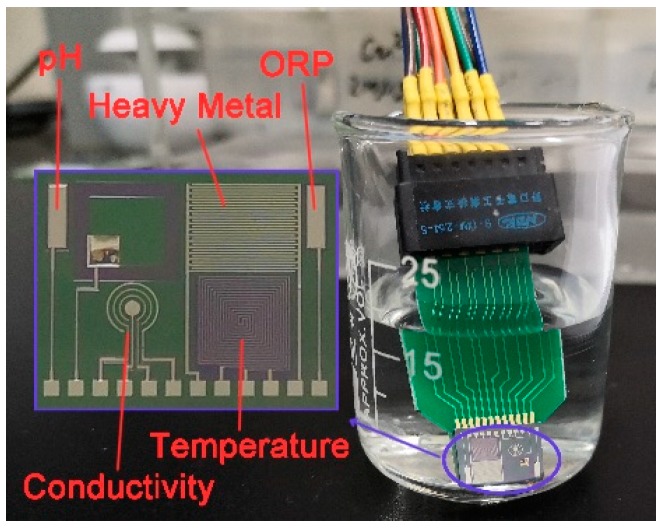
Multi-parameter integrated chip.

Before used, the silicon wafers were boiled in a concentrated H_2_SO_4_/H_2_O_2_ mixture and deionized water at 2000 °C for 20 min successively. Then, the wafers were sequentially put into acetone, alcohol, and deionized water for ultrasonic cleaning. Since silicon has electrical conductivity, it is necessary to perform an oxidation treatment on the silicon surface for insulation. The SiO_2_ layer formed by thermal oxidation was 600–900 nm thick (Figure 1 Step 1). The surface of the SiO_2_ layer was then cleaned using plasma of oxygen. We used photolithography to make the required sensor shape patterns appear on the wafer. After being cleaned by oxygen plasma, metal Ti and Pt were sputtered on the position of the sensors on the surface of the wafer by magnetron sputtering technology (Figure 1 Step 2). The Ti performed as bonded metal to enhance the adhesion of Pt to the substrate. Excess metal and photoresist were removed with acetone by lift-off processes. Repeated the above steps to get a layer of metallic Ag for future use (Figure 1 Step 3). Then a photoresist insulating layer of SU8 was prepared around the sensor electrodes metal layer also by photolithography technology (Figure 1 Step 4). Subsequently, the silicon wafer was sliced to form a batch of single chips of 8 mm × 10 mm. The integrated chip of multi-parameter water quality sensors is shown in Figure 2. Each chip was connected to the PCB board by golden wire ball welding finally. 

The pH electrode and ORP electrode are rectangular in shape and 500 μm × 2000 μm in size. The basic principles of the two parameters are similar and both based on the potential detection of the Nernst equation as described in Equation (1).

(1) $${E\;=\;E}_{0}\;+\;\frac{RT}{nF}ln\frac{A_{ox}}{A_{red}}$$

where *E* is the potential generated on the surface of metal electrodes. E_0_ is the standard electrode potential (mV). R is the ideal gas constant (8.314 J·mol^−1^·K^−1^). F is the Faraday constant (96,485 C/mol). The symbol *n* represents the number of electron transfers in the reaction. *A_ox_* represents the activity of oxidizing substances (mol/L) and *A_red_* represents the activity of the reducing substance (mol/L). Obviously, the potential *E* we need to measure is affected by the thermodynamic temperature *T* (K). The measuring electrode of the ORP sensor is an inert Pt electrode, which can play the role of electron transfer and does not participate in the reaction. Therefore, ORP is measured directly by detecting the potential of Pt microelectrode against an Ag/AgCl reference electrode as a single voltage in millivolts. The Pt measuring cell detects changes in ORP, while the RE provides a stable comparison signal.

RuO_x_ is sensitive to the pH of the solutions. After deposited a RuO_x_ layer on the surface of Pt microelectrode by cyclic voltammetry (CV) in RuCl_3_ solution, the RuO_x_/Pt electrode turns into a pH sensor combined with the Ag/AgCl reference electrode. The simplified induction mechanism of RuO_x_ [27] can be described by Equation (2). The Nernst equation of RuO_x_ is reduced to Equation (3).

(2) $${\mathrm{Ru}}^{\mathrm{IV}}O_{2}{\;+\;e}^{-}{\;+\;H}^{+}\;⇌{\;\mathrm{Ru}}^{\mathrm{III}}O\left(\mathrm{OH}\right)$$

(3) $$E_{\mathrm{pH}}{\;=\;E}_{0}-\frac{RT}{F}\ln\frac{c\left({\mathrm{Ru}}^{\mathrm{III}}\right)}{c\left({\mathrm{Ru}}^{\mathrm{IV}}\right)\cdot c\left(H^{+}\right)}$$

The *c*(Ru^III^) and *c*(Ru^IV^) represent the activities of Ru(III) and Ru(IV), respectively. In the solid state, they are nearly equal. The *c*(H^+^) is the concentration of H^+^ and its negative logarithm is defined as pH. At 25 °C, it is approximately Equation (4).

(4) $$E_{\mathrm{pH}\;}{=\;E}_{0}+0.05914\times \mathrm{lg}c\left(H^{+}\right)$$

That means the theoretical slope is −59.14 mV/pH. The same as ORP, the pH sensing electrode measures the open circuit potential (OCP).

The conductivity sensor was designed as a four-electrode type. The advantage of the 4-electrode conductivity sensor lies in the fact that there is negligible current flowing through the inner electrodes where the measurement is made [12]. Conductivity is the inverse of resistivity. It is an electrical physical quantity, and its relationship with voltage and current can be described as Equation (5).

(5) S = k⋅G = k⋅I / U

where *S* is the conductivity (mS/cm), k the electrode constant (cm^−1^), *G* the conductance (mS), *I* the current (μA), and *U* the voltage (mV). The detection method is applying an AC stimulus to the sensor and measuring the voltage and current across it. Based on the experimentally measured voltage, current, and electrode constant, the electrical conductivity of the water quality can be calculated. Also, conductivity is affected by temperature as Equation (6).

(6) $$S_{t}{\;=\;S}_{18}\cdot \left[\;\alpha \left(t-18\right)\;+\;1\;\right]$$

where *S_t_* is the conductivity (mS/cm) at *t* °C and *S*_18_ means the conductivity (mS/cm) at 18 °C. The symbol α is the temperature coefficient (°C^−1^).

As stated previously, RTD is widely used to measure temperature. Its resistance-temperature characteristic can be expressed as Equation (7).

(7) $$R_{t}{\;=\;R}_{0}\cdot \left[\;1\;+\;\beta \left(t\;-{\;t}_{0}\right)\;\right]$$

where *R_t_* means the resistance (Ω) at *t* °C and R_0_ means the resistance (Ω) at t_0_ °C. And β is the temperature coefficient (°C^−1^) of the material. The method of measuring resistance is simple. The temperature sensor in this study is a Pt microband with an orthogonal structure. The connection adopts a three-terminal connection method [4,12].

For copper ions detection, an electrochemical 3-electrode system was built in this work. Two rows of interdigital electrodes were designed as WE and CE respectively. Each row of the electrodes consisted of 15 finger electrodes (3500 μm × 50 μm) with the interdigital spacing of 50 μm. A film of AuNPs was deposited on the surface of WE in 2 mM HAuCl_4_ solution by cyclic voltammetry. The deposition potential was −0.2 V and the deposition time was 300 s [23]. In this 3-electrode system, an Ag/AgCl electrode was used as RE.

### 2.2. Hardware System

**Figure 3:**
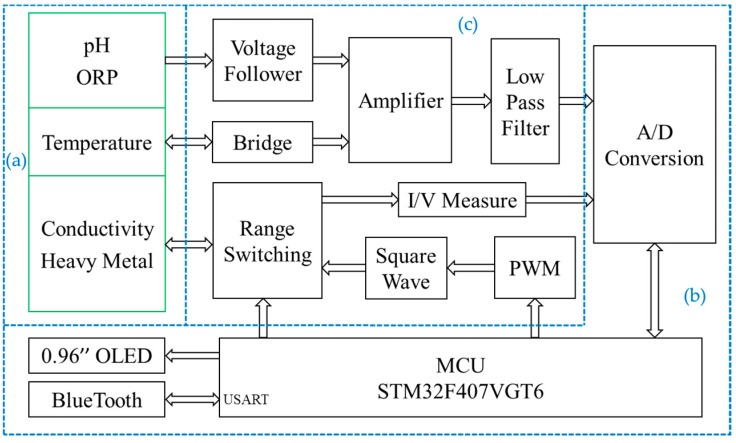
Hardware system block diagram: (**a**) integrated sensor chip; (**b**) control module, human-computer interaction module and A/D conversion (control board); (**c**) signal conditioning circuit (detection board).

The detection system consists of an integrated sensor chip as described above, a control module, a human-computer interaction module, analog-to-digital (A/D) conversion, and signal conditioning circuit. The hardware block diagram of the detection system is shown in Figure 3.

The control module adopts a piece of STM32F407VGT6 chip (STMicroelectronics, Geneva, Switzerland) as the main controller and contains the smallest system composed of the external crystal oscillator, debugging interface, reset circuit and so on. The human-computer interaction module consists of a 0.96-inch OLED screen, a Bluetooth unit (BLE103 chip, Wenheng Technology, Shanghai, China) [28] and a serial communication port of USART. The OLED screen can display real-time data, which is used to prompt the running state of the system and display the measurement results directly. By using Bluetooth communication, we can connect the portable water quality detection system with a smartphone to achieve functional control, data transmission, and analysis. In order to store large amounts of data locally, we use the USART port to transmit data to the computer. Data processing and result curve drawing are performed by the Origin software (Version 9.6.5, OriginLab, Northampton, MA, USA). A low power analog-to-digital converter (ADC) chip AD7790 (Analog Devices, Norwood, MA, USA) is used in the measurement. Under the reference voltage of 2.5 V, it can obtain the smallest measurement of 0.0763 mV [29]. When the pH sensor acts as −59.14 mV/pH, the smallest theoretical change for pH we can detect is 0.0763/59.14 = 0.0013 pH. In the detection system, only two decimal places are retained to achieve a resolution of 0.01 pH.

**Figure 4:**
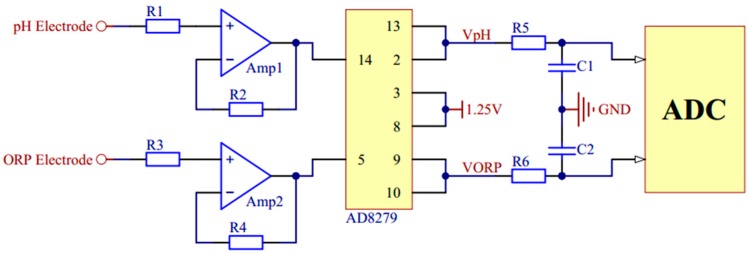
The signal conditioning circuit of pH and oxidation-reduction potential (ORP).

The signal conditioning circuit mainly includes filters, operational amplifiers, and the signal generator circuit. For the OCP measurement of pH and ORP, an AD8279 chip (Analog Devices, Norwood, MA, USA) is used to amplify or reduce their voltage by two times. Then it adds the compensation voltage of 1.25 V to get the measurement voltage in 0–2.5 V. Considering impedance matching, two voltage followers are used. The signal conditioning circuit of pH and ORP is shown in Figure 4. The RE of them is connected to a zero potential of GND through a resistor.

**Figure 5:**
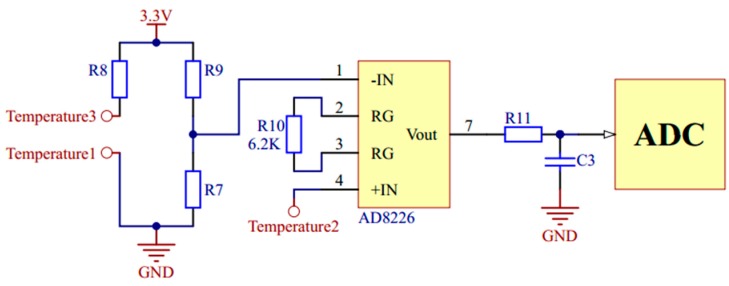
The signal conditioning circuit of temperature.

For temperature measurement, the Wheatstone bridge is used to convert the change of resistance value to voltage change. The voltage is amplified by using a single resistance amplifier AD8226 (Analog Devices, Norwood, MA, USA). The signal conditioning circuit of temperature is shown in Figure 5.

**Figure 6:**
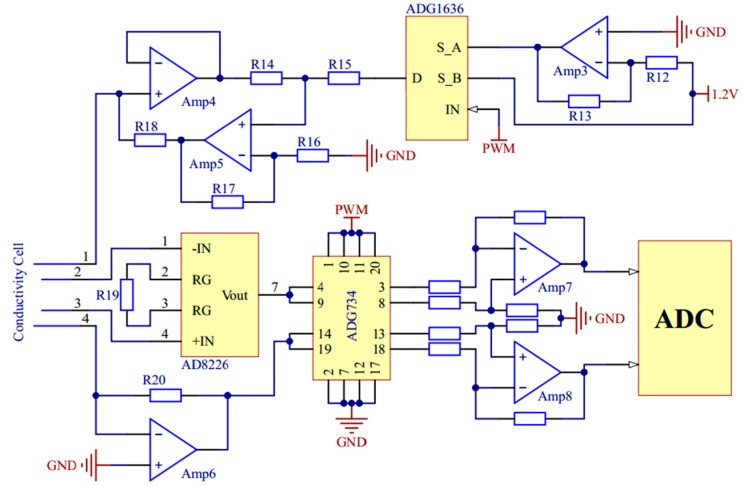
The signal conditioning circuit of conductivity.

**Figure 7:**
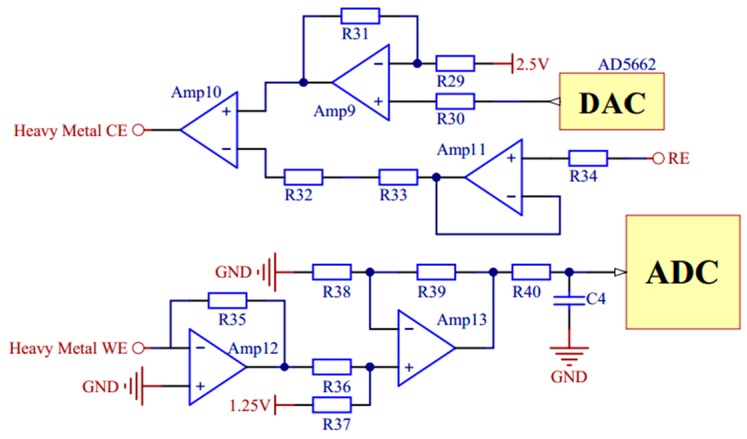
The signal conditioning circuit of copper irons.

In the measurements of conductivity and copper irons, voltage excitation of specific frequency and amplitude is needed. A pulse width modulation circuit and a digital-to-analog converter (DAC) chip AD5662 (Analog Devices, Norwood, MA, USA) are used respectively to generate bipolar square wave and variable pulse voltage. The signal conditioning circuits of conductivity and copper irons are shown in Figure 6 and Figure 7.

**Figure 8:**
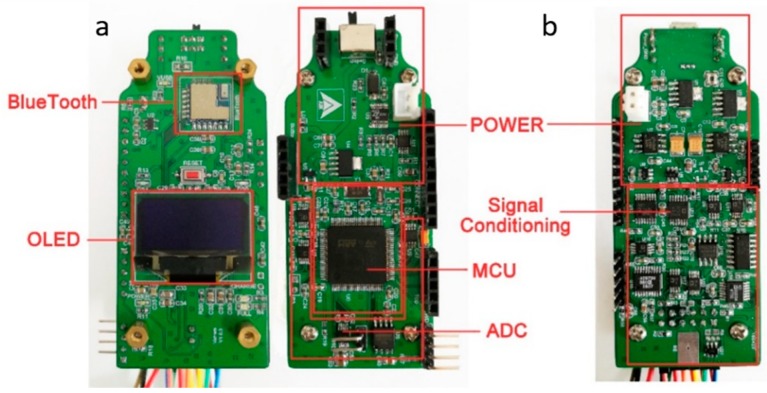
The circuit boards of the portable detection system: (**a**) Control board with both sides; (**b**) Detection board with the top side.

Besides, the whole system also has a complete power management module to provide a stable and continuous power supply for all hardware components. The external power supply source is a lithium battery with a capacity of 1000 mA·h. Different precise voltages of ±3.3 V, 1.25 V, and 2.5 V are obtained through a series of low dropout regulator chips. The circuit boards of the portable detection system are shown in Figure 8. According to the functions in Figure 3, all the chips and devices are placed on a control board and a detection board.

### 2.3. Software System

**Figure 9:**
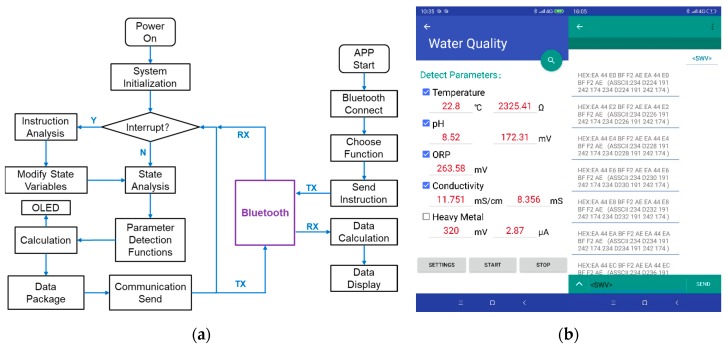
The software system: (**a**) Flowchart of the embedded software and application (APP) software; (**b**) A simple UI and the background data of the APP.

The embedded software program of the main controller was written in C language based on the Keil software integrated development environment. It mainly realizes the functions of hardware driver management, detection function selection, data calculation, and communication. A simple application (APP) was developed on the software Android Studio in Java language. The flowchart of the whole software system including embedded software and APP software program is shown in Figure 9a. A simple user interface (UI) and the background control instruction sending with data stream receiving of the APP [28] on the smartphone are shown in Figure 9b.

In the program, the detection of each parameter is encapsulated into each function. There are time interrupts and serial port interrupts in the MCU. In the interrupt service program, the instructions received from the serial port Bluetooth will be analyzed. The instructions are transmitted as char strings. On the mobile side, the instructions are sent by checkbox or text input. The instructions have a fixed format and a set of contents, which must be enclosed in angle brackets. Different contents of the instructions have different function commands. C language can analyze char strings easily and choose the needed program functions for multi-parameter detection. The detection data is also transmitted by the serial port. The data strings are encoded in ASCII. In this paper, through the serial port, a large amount of experimental data is sent to the computer besides the mobile phone. For the convenience of drawing the resulting diagram, the software Origin (Version 9.6.5, OriginLab, Northampton, MA, USA) is used on the computer.

For the OCP detection of voltage such as pH and ORP, we need to read the data in ADC chip, and then calculate the value of physical quantity. For temperature detection, the design of the hardware has already used the Wheatstone bridge to convert the resistance into voltage. The program reads the ADC and calculates it. According to the relationship between platinum thermal resistance and temperature in Equation (7), the temperature measurement can be obtained after the calibration process. In this paper, a 10 kHz bipolar square wave is used to measure the conductivity, which can eliminate the influence of the capacitance between electrodes [12]. Using the pulse width modulation function of the MCU, a 10 kHz unipolar square wave can be generated, and then the required bipolar excitation is obtained by the voltage inverter in the hardware circuit. The program controls reading ADC values while the square wave is applied. The measured conductivity value is obtained by voltage-ampere conversion and Ohm’s law calculation. According to the calibrated electrode constant, the conductivity can be computed.

Common electrochemical methods for heavy metal ions detection are differential pulse voltammetry, linear sweep voltammetry and square wave voltammetry (SWV) [30,31]. Among them, SWV has higher sensitivity, wider linear range and low detection limit according to the report [31]. In our study, SWV is used in the detection of copper ions. By analyzing the position and height of the dissolution peak in the voltage-current diagram, the concentration of copper ions is obtained [30]. In the software program, the value of pulse voltage is changed in each fixed interruption period, and the average values of the top and bottom of a single periodic pulse are taken as the data points in the volt-ampere curve. Similar to SWV, the function of the CV process is added to the software. The CV is mainly used to profile the electrochemical characteristics of the microelectrodes [4,23,31].

## 3. Results and Discussion

**Figure 10:**
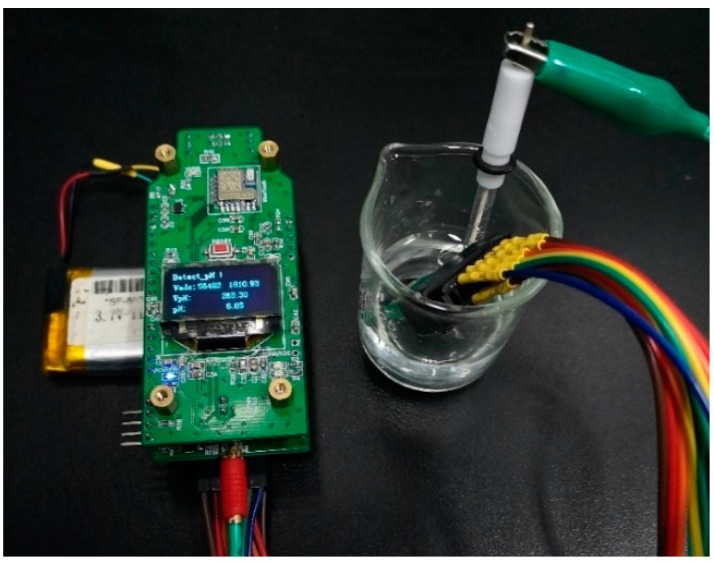
A photograph of the actual measurement.

According to the aforementioned principles and equations (1,4–7), we carried out calibration experiments of the 5 parameters (pH, temperature, ORP, conductivity, and Cu^2+^) using our portable detection system and chip. Except for the temperature calibration, all other experiments were performed at room temperature. The sensor chip was washed with deionized water between each measurement and dried in the flowing air. A photograph of the actual measurement is shown in Figure 10.

### 3.1. pH and Temperature Calibration Measurements

**Figure 11:**
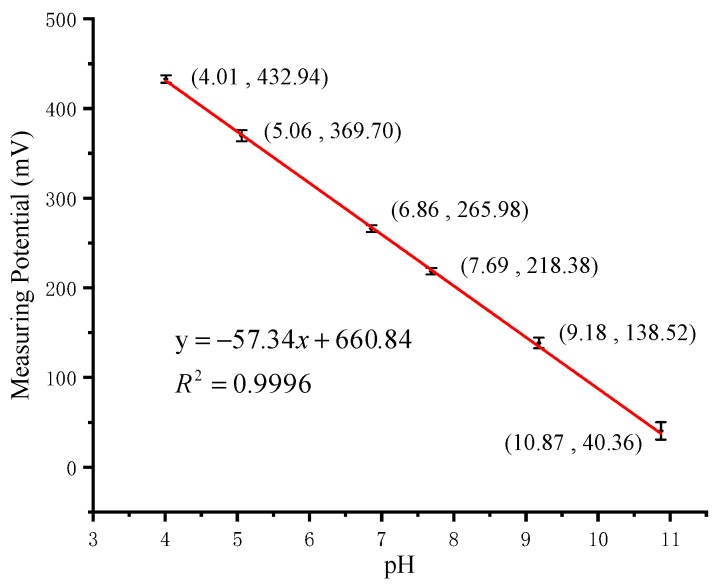
Calibration curve of pH sensor.

Six standard solutions were prepared for pH measurement in the range of 4–11. The standard solutions consisted of 0.2M NaOH in different volumes added to the Britton Robinson buffer [12]. To ensure the correct configuration of the standard solutions, a pH meter (PHS-3C, INESA Scientific Instrument, Shanghai, China) with a glass electrode (E-201-9, Ruosull Technology, Shanghai, China) was used to measure the pH. The results were used as the x-axis data of the calibration curve. The calibration measurement started with the minimum standard solution (pH = 4.01) and increased. The time for the system to stabilize in each solution was within 30 s. Three repeated tests from 4.01 to 10.87 were carried out on our portable detection system. The calibration curve results are shown in Figure 11. The sensor shows a super-Nernst response of pH and has a sensitivity of −57.34 mV/pH, which is close to the theoretical value −59.14 mV/pH in Equation (4). The previously reported sensitivity −62.88 mV/pH of this kind of sensor was detected by an electrochemical workstation of GAMRY Reference 600 [12]. The result shows that our portable detection system has satisfactory detection capabilities. In the calibration curve, the span is calculated as 10.87−4.01=6.86. The maximum absolute error of pH measurement is 0.07 when pH = 9.18. The accuracy is computed as 0.07/6.86=1.02%.

**Figure 12:**
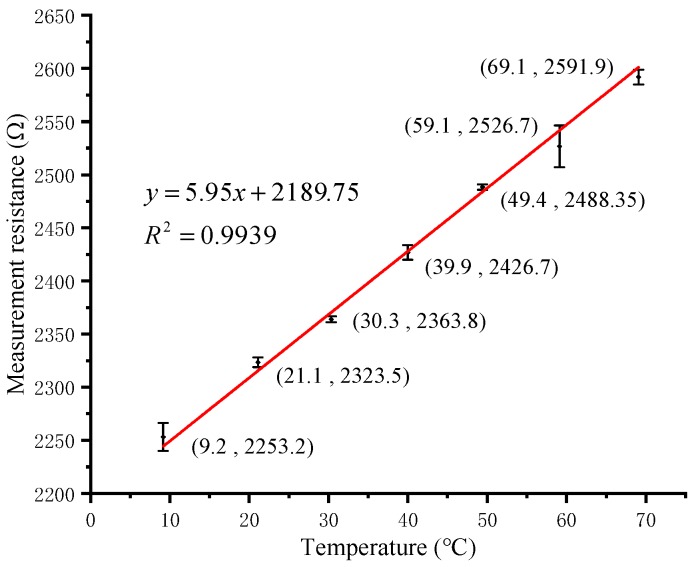
Calibration curve of temperature sensor.

Temperature measurements were carried out using a water bath heater (HH-1, Kewei Yongxing Instrument, Beijing, China) to steady the temperature and measured every 10 °C in the range of 10–70 °C for three times. The heated and measured solutions were fresh ice-water mixtures. We used an external metal cylindrical thermometer (Pt100, Sanxing Temperature Meter, Dongyang, China) as the standard rather than the set temperature of the heater, which could avoid control errors of the heater. The calibration curve is shown in Figure 12. The three-wire orthogonal Pt thermal resistor sensor has a temperature response capacity of 5.95 Ω/°C. For unknown solutions to be detected, using the portable system to measure the resistance of the RTD, the program can calculate the temperature by the equation in the calibration curve and display it on the OLED screen. In Figure 12, the maximum absolute error is 2.4 °C when the temperature is 59.1 °C. So the accuracy of temperature measurement is 2.4/(69.1−9.2) = 4.01%.

### 3.2. ORP and Conductivity Calibration Measurements

**Figure 13:**
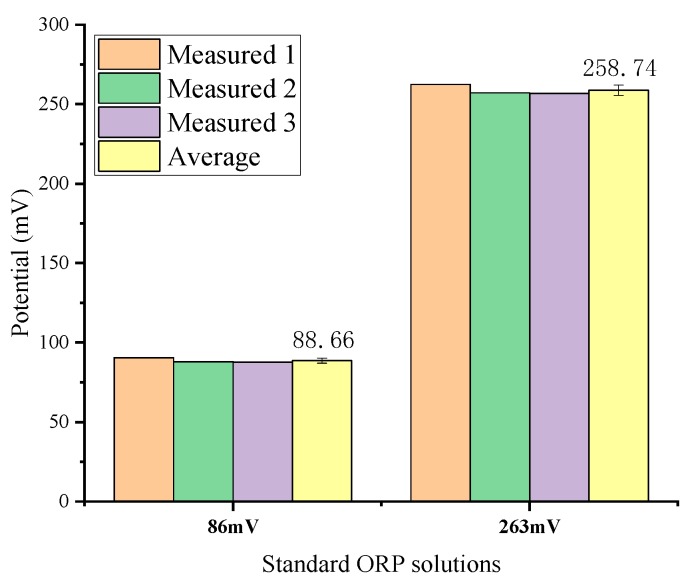
ORP detection result.

Two standard solutions of 86 mV (quinhydrone solution with pH = 7) and 263 mV (quinhydrone solution with pH = 4) were used for ORP measurements. The RE was the Ag/AgCl glass electrode. The average values obtained by three measurements are 88.66 mV and 258.74 mV respectively as shown in Figure 13. The relative error in the detection of the ORP sensor is less than 3%, which shows good precision and repeatability. Commercial ORP instruments typically use one-point calibration as standardization [32,33]. The standard solution can be a Light’s solution (476 mV) or a ZoBell’s solution (229 mV) [32].

**Figure 14:**
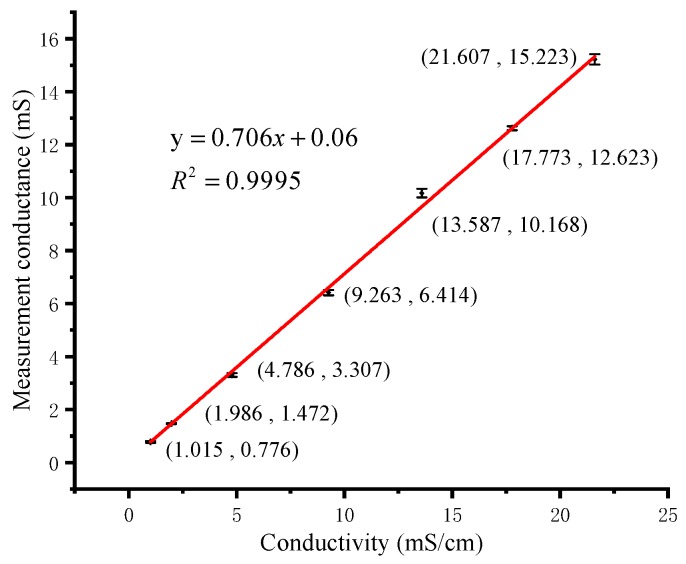
Calibration curve of conductivity sensor.

A bipolar square wave at 10 kHz frequency was selected to excite the four-electrode ring conductivity sensor in our detection system. The sensor was calibrated with 7 standard solutions in the range of 1–22 mS/cm. These standard solutions were pure NaCl solutions with different mass percentage concentrations of 0.05, 0.10, 0.25, 0.50, 0.75, 1.00 and 1.24, respectively. At constant temperature, the fixed mass fraction solution has fixed conductivity [34]. A commercial portable conductivity meter (8306, AZ Instrument, Taichung, China) was used to measure the conductivity of these standard solutions at room temperature. The calibration curve of conductivity detection is shown in Figure 14. The electrode constant of the conductivity sensor is calculated as 1/0.706=1.416 cm^−1^ from the three sets of volt-ampere characteristic data obtained by the portable meter. The experimental result shows that the four-electrode conductivity sensor has good linearity and sensitivity. When measuring unknown solutions, we first use the system to measure conductance and temperature values. Then the point of the conductance is substituted into the calibration curve in the detection program, and the conductivity of the solution is calculated using Equation (6). The measuring range of conductivity is 1.015–21.607 mS/cm. The maximum absolute error is 0.730 mS/cm, which includes calculation errors. The accuracy is computed as 0.730/(21.607−1.015)=3.55%.

### 3.3. Copper Ions Measurement

**Figure 15:**
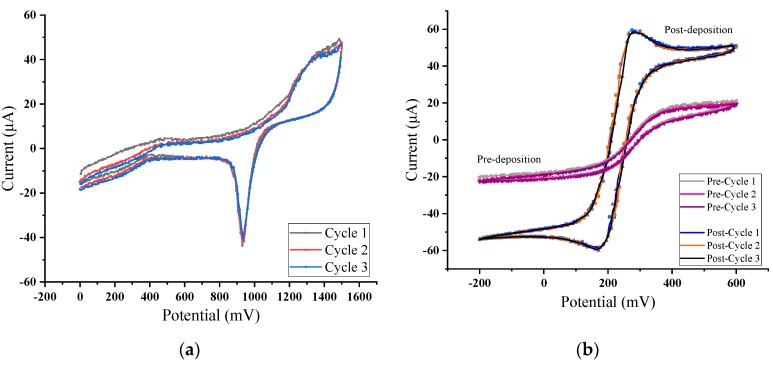
Cyclic voltammetry (CV) characteristic curves in: (**a**) H_2_SO_4_; (**b**) K_3_[Fe(CN)_6_].

In order to test the feasibility of the system for the detection of heavy metal ions in water, copper ions were chosen as the typical ions. The buffer solution was HAC-NaHAC solution with pH = 4.5. Four standard solutions with different copper ions concentrations (0.0, 0.2, 0.4, 0.6 mg/L) were used as the calibration solutions. To improve the detection sensitivity, a film of AuNPs was electrodeposited on the surface of the interdigital working electrode. The deposition was set at the potential −0.2 V in 2 mM HAuCl_4_ solution for 300 s. Figure 15 shows the CV characteristic curves of the interdigital electrodes in H_2_SO_4_ and K_3_[Fe(CN)_6_] solutions after deposition.

As can be seen from Figure 15b above, the deposition of AuNPs can improve the redox characteristics of the interdigital electrodes. SWV scanning tests were carried out in copper ion solutions with different concentrations. The parameters of SWV are shown in Table 1.

**Figure 16:**
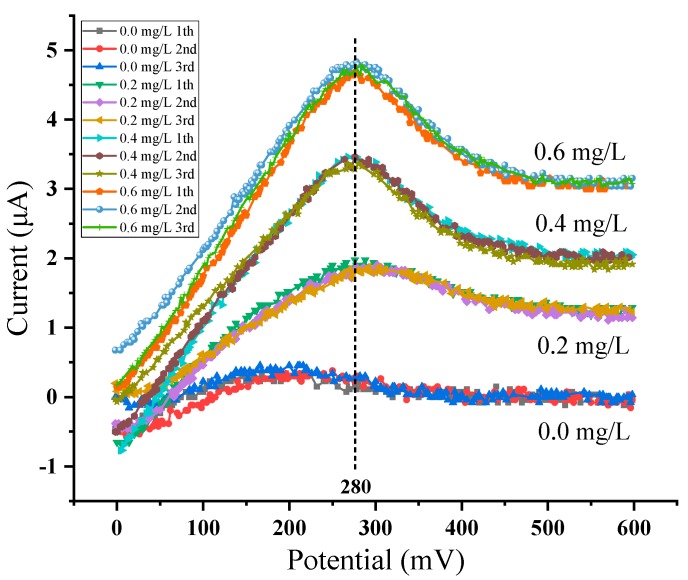
Cu^2+^ concentration-response curve.

**Figure 17:**
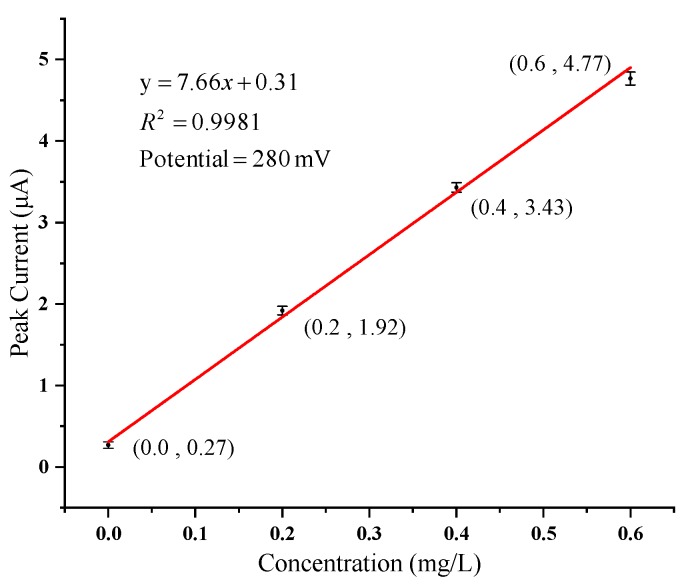
Cu^2+^ concentration-current fitting line.

The response curves of the sensor system to different concentrations of copper ion solutions are shown in Figure 16, in which only the current response ranging from 0–600 mV is shown. The order from bottom to top is 0.0, 0.2, 0.4, 0.6 mg/L. The peak potential of copper ion dissolution is 280 mV when the working electrode is a platinum interdigital electrode modified with AuNPs. The average value of each concentration is taken as the final peak current value at least three times after detection. The relationship between concentration and peak current is recorded and linear fitting is made as shown in Figure 17. The linearity for fitting a straight line is 0.9981.

The standard GB/T 5750-2006 [26] defines the detection limit as Equation (8).

(8) $$D_{L}\;=\;2\sqrt{2}{\;t}_{f}{\;S}_{wb}$$

where t_f_ is the value of t at a significance level of 0.05 and degrees of freedom f. The f is obtained by subtracting one from the number of repeated detections. For three repeated measurements, the f is 2. In the statistical table, t_2_ is 2.92. *S*_wb_ is the standard deviation of the blank sample. In this experiment of Cu^2+^, the standard deviation measured in the concentration of 0.0 mg/L is 0.04. According to Figure 17 and Equation (8), the detection limit of Cu^2+^ concentration is computed as 2.33 μg/L.

## 4. Conclusions

In this paper, a multi-parameter integrated sensor chip fabricated by MEMS technology is further studied, on which five different sensors are distributed, and its portable system for water quality detection is developed. Calibration experiments for all sensors were performed using the portable system. The pH sensor electrode fabricated by electrodepositing RuO_x_ sensing material on the surface of the Pt electrode has a sensitivity of −57.34 mV/pH. The measuring range is 4.01–10.87 pH and the system presents an accuracy of 1.02%. ORP is directly detected by the Pt microelectrode and the relative error is less than 3%. The Pt thermistor with a three-wire orthogonal structure has a temperature response of 5.95 Ω/°C. In the measuring range of 9.2–69.1 °C, the accuracy of temperature is calculated as 4.01%. In the detection of conductivity solution in the range of 1–22 mS/cm, the sensor has an electrode constant of 1.416 cm^−1^ and the linearity is 0.9995, which shows an excellent linear detection performance of the sensor and the precise control of detection abilities. As to the detection of copper ions, AuNPs were deposited on the working electrode to improve the performance of the sensor. The peak current of the sensor shows a good linearity to the concentration of Cu^2+^ in the range of 0–0.6 mg/L. The detection limit of Cu^2+^ concentration is 2.33 μg/L. The experimental results show that this multi-parameter sensor chip and its portable system have the potential for on-site detection of various water quality parameters.

## Figures and Tables

**Table 1:** Square wave voltammetry (SWV) parameters setting.

Accumulation Time	Accumulation Potential	Step	Frequency	Initial Voltage	Final Voltage	Pulse Height
300 s	−600 mV	2 mV	25 Hz	−100 mV	800 mV	25 mV
